# Transgenic Expression of FoxM1 Promotes Endothelial Repair following Lung Injury Induced by Polymicrobial Sepsis in Mice

**DOI:** 10.1371/journal.pone.0050094

**Published:** 2012-11-20

**Authors:** Xiaojia Huang, You-Yang Zhao

**Affiliations:** 1 Department of Pharmacology, University of Illinois College of Medicine, Chicago, Illinois, United States of America; 2 Center for Lung and Vascular Biology, University of Illinois College of Medicine, Chicago, Illinois, United States of America; Ottawa Hospital Research Institute, Canada

## Abstract

Enhancing endothelial barrier integrity for the treatment of acute lung injury (ALI) is an emerging novel therapeutic strategy. Our previous studies have demonstrated the essential role of FoxM1 in mediating endothelial regeneration and barrier repair following lipopolysaccharide-induced lung injury. However, it remains unclear whether FoxM1 expression is sufficient to promote endothelial repair in experimental models of sepsis. Here, employing the FoxM1 transgenic (*FoxM1 Tg*) mice, we showed that transgenic expression of FoxM1 promoted rapid recovery of endothelial barrier function and survival in a clinically relevant model of sepsis induced by cecal ligation and puncture (CLP). We observed lung vascular permeability was rapidly recovered and returned to levels similar to baseline at 48 h post-CLP challenge in *FoxM1 Tg* mice whereas it remained markedly elevated in WT mice. Lung edema and inflammation were resolved only in *FoxM1 Tg* mice at 24 h post-CLP. 5-bromo-2-deoxyuridine incorporation assay revealed a drastic induction of endothelial proliferation in *FoxM1 Tg* lungs at 24h post-CLP, correlating with early induction of expression of FoxM1 target genes essential for cell cycle progression. Additionally, deletion of FoxM1 in endothelial cells, employing the mouse model with endothelial cell-restricted disruption of *FoxM1* (*FoxM1 CKO*) resulted in impaired endothelial repair following CLP challenge. Together, these data suggest FoxM1 expression in endothelial cells is necessary and sufficient to mediate endothelial repair and thereby promote survival following sepsis challenge.

## Introduction

The endothelial monolayer lining the inner wall of blood vessels controls the transvascular flux of fluid, proteins, and cells across the vessel wall into underlying tissue [1]–[3]. Intractable endothelial injury characterized by persistently increased lung microvascular permeability resulting in protein-rich lung edema is a hallmark of acute lung injury (ALI) and its severe form acute respiratory distress syndrome (ARDS) [4]–[6]. Enhancing endothelial barrier integrity for the treatment of ALI/ARDS is a previously unappreciated but emerging novel therapeutic strategy [7], [8]. Hence, it is important to delineate the molecular mechanisms regulating endothelial repair following lung vascular injury.

FoxM1 is a member of the mammalian fox family of transcription factors that share homology in their winged helix DNA-binding domains [9]–[11]. FoxM1 is expressed in proliferating cells including cancer cells, where it controls cell cycle progression into DNA replication (G1/S) and mitosis (G2/M), and silenced in terminally differentiated cells [12]–[15]. FoxM1 is essential for transcription expression of the S-phase kinase-associated protein 2 and Cdk subunit 1 to regulate the degradation of Cdk inhibitor proteins p21^Cip1^ and p27^Kip1^ during the G1/S transition [13]. FoxM1 also controls the transcription of genes critical for G2/M and mitotic progression including cyclin B1, Cdc25B and Cdc25C phosphatases, polo-like kinase 1 and aurora kniase [13], [14]. FoxM1 transcriptional activity requires phosphorylation at Thr596 by either the S-phase or M-phase Cdk-cyclin complexes and subsequent recruitment of p300/CBP coactivator proteins [16].

In response to various stimuli, FoxM1 expression is induced in several cell types *in vivo* including hepatocytes and lung epithelial cells and plays an important role in liver regeneration and alveolar repair, respectively [13], [17]. FoxM1 expression is also markedly induced in the pulmonary vascular endothelial cells (EC) following lipopolysaccharide (LPS) challenge [18]. Intriguingly, FoxM1 is only induced during the recovery phase following LPS challenge. Employing the mouse model with EC-restricted disruption of *FoxM1* (*FoxM1 CKO*), we have shown the critical role of FoxM1 in regulating endothelial proliferation and endothelial repair following lung vascular injury induced by LPS challenge [18]. *FoxM1 CKO* mice exhibit persistent lung vascular leakiness and increased mortality following LPS challenge. We have also shown that FoxM1 is essential for re-annealing of endothelial adherens junction complex and thereby restoration of endothelial barrier integrity through transcriptional control of β-catenin expression [19]. β-catenin is the integral protein of adherent junctions [20], [21]. However, it remains unclear whether FoxM1 expression is sufficient to promote endothelial repair following lung injury. Especially, it is unknown if FoxM1 is critical for endothelial repair following polymicrobial sepsis induced by cecal ligation and puncture (CLP), a well-recognized clinically relevant rodent model of sepsis [22]–[24]. Here, employing *FoxM1* transgenic mice (*FoxM1 Tg*) as well as *FoxM1 CKO* mice, we show that FoxM1 expression is necessary and sufficient to promote endothelial regeneration and barrier repair following lung injury induced by CLP challenge.

## Materials and Methods

### Mice

FoxM1 transgenic mice were obtained from Dr. Robert H. Costa at the University of Illinois College of Medicine [25]. *FoxM1 CKO* mice were previously made in our laboratory (18, 19). All mice were bred and maintained in the Association for Assessment and Accreditation of Laboratory Animal Care-accredited animal facility at the University of Illinois at Chicago according to National Institutes of Health guidelines. All animal experiments were performed in accordance with protocols approved by the University of Illinois at Chicago Animal Care and Use Committee.

For survival study, mice following CLP or sham operation had normal access for water and hood, and were monitored four times a day over the course of 7 days. Moribund animals were identified by labored breathing pattern defined as a decreasing rate of respiration and/or an inability to ambulate in response to stimulation. Moribund mice were euthanatized using CO2 followed by cervical dislocation. At the end of the study (day 7), all the survived mice were euthanatized with CO2 followed by cervical dislocation.

### Sepsis Models

CLP was performed as previously described [22], [24]. Briefly, mice were anesthetized with isoflurane, and then a 1-cm midline abdominal incision was made. The cecum was identified, ligated and punctured with a 21-gauge needle. A small amount of cecal content was extruded to ensure the patency of injury. The cecum was returned to the abdominal cavity. Sham-operated mice were treated with cecal manipulations but without ligation and puncture.

LPS (Sigma-Aldrich, St. Louis, MO) at 7.5 mg/kg BW was administered by i.p. injection to induce sepsis.

### Vascular Permeability Assessment

The Evans Blue-conjugated albumin (EBA) extravasation assay was performed as previously described [26]. EBA at a dose of 20 mg/kg BW was retroorbitally injected into mice 30 minutes before tissue collection. Lungs were perfused free of blood with PBS, blotted dry, and weighed. Lung tissue was homogenized in 1 ml PBS and incubated with 2 volumes of formamide at 60°C for 18 hours. The homogenate was then centrifuged at 5,000 × g for 30 minutes. The optical density of the supernatant was determined at 620 nm and 740 nm. The extravasated EBA in lung homogenate was expressed as µg of Evans Blue dye per g lung tissue.

### Myeloperoxidase (MPO) Assay

MPO activity was measured as previously described [18], [27]. Briefly, Lung tissues were collected following perfusion free of blood with PBS and homogenized in 50 mM phosphate buffer. Homogenates were centrifuged at 15,000 ×g for 20 minutes at 4°C. Thereafter the pellets were resuspended in phosphate buffer containing 0.5% hexadecyl trimethylammonium bromide (Sigma-Aldrich, St Louis, MO) and subjected to a cycle of freezing and thawing. Subsequently the pellet was homogenized and the homogenates were centrifuged again. The supernatants were assayed for MPO activity using kinetics readings for 3 min and absorbance was measured at 460 nm. The results were presented as ΔOD_460_/min/g lung tissue.

### Histological Analysis

Following PBS perfusion, the lung tissues were fixed for 5 min by instillation of 10% PBS-buffered formalin through trachea at a trans-pulmonary pressure of 15 cm H_2_O. After tracheal ligation, the lungs were fixed with 10% PBS-buffered formalin overnight at 4°C. After paraffin embedding process, the tissues were sectioned at 5 µm thick and stained with H & E.

### Cell Proliferation Assay

5-bromo-2-deoxyuridine (BrdU, Sigma-Aldrich, St Louis, MO) was administered by i.p. injection into mice (75 mg/kg BW) 4 h prior to tissue collection [18]. Mouse lung cryosections (5 µm) were stained overnight with anti-BrdU (1∶3, BD Biosciences, San Jose, CA), and incubated with Alexa Fluor 488-conjugated secondary antibody (1∶200, Life Technologies, Grand Island, NY). Lung vascular endothelial cells were immunostained with anti-vWF (1∶300, Sigma-Aldrich, St. Louis, MO) and anti-CD31 (1∶40, Abcam, Cambridge, MA) antibodies at 4°C. Then the sections were incubated with Alexa Fluor 594-conjugated secondary antibody (1∶200, Life Technologies, Grand Island, NY). The nuclei were counterstained with DAPI (Life Technologies, Grand Island, NY).

### Molecular Analysis

Total RNA was isolated using an RNeasy Mini kit including DNase I digestion (Qiagen, Valencia, CA). Then one-step RT-PCR analysis was performed with a sequence detection system (ABI Prism 7000; Life Technologies, Grand Island, NY) with a SYBR Green 1-step kit (Life Technologies, Grand Island, NY). The following primer sets were used for analyses: mouse FoxM1 primers, 5′-CACTTGGATTGAGGACCACTT-3′ and 5′-GTCGTTTCTGCTGTGATTCC-3′; and mouse cyclophilin primers, 5′-CTTGTCCATGGCAAATGCTG-3′ and 5′-TGATCTTCTTGCTGGTCTTGC-3′. Primers for mouse Cdc25 C, cyclin B1, cyclin F, cyclin A2, TNF-α, MIP-2, IL-6 and ICAM-1 were purchased from Qiagen. The mouse gene expression was normalized to cyclophilin.

Western blot analysis was performed using an anti-FoxM1 or anti-Cdc25C antibody (1∶500, Santa Cruz Biotechnology, Santa Cruz, CA). The same blots were re-probed with an anti-β-actin antibody (1∶3000, BD Biosciences, San Jose, CA) as a loading control.

### Statistical Analysis

Differences between groups were examined for statistical significance using Student’s *t*-test or *ANOVA* with Bonferroni correction. A *P* value less than 0.05 was considered significant. Significance in survival studies was examined with Log-Rank (Mantel-Cox) test.

## Results

### Rapid Recovery of Vascular Integrity in *FoxM1 Tg* Mice Following CLP Challenge

**Figure 1:**
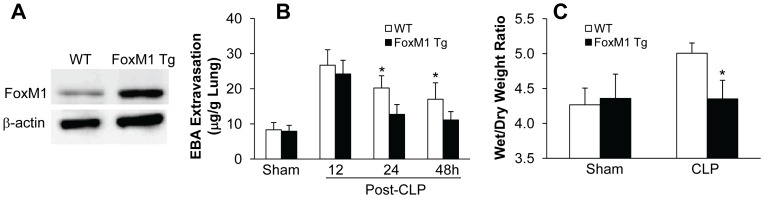
Rapid recovery of vascular permeability in *FoxM1 Tg* lungs following CLP challenge. (**A**) Representative Western blotting demonstrating increased expression of FoxM1 in *FoxM1 Tg* lungs. Thirty µg of lung lysates were loaded per lane. FoxM1 expression was detected with anti-FoxM1 antibody. The same membrane was immunoblotted with an anti-β-actin antibody for loading control. (**B**) Lung vascular permeability assessed by EBA extravasation assay. Various times following CLP challenge, mouse lung tissues were collected for EBA assay. Lung tissues from sham-operated mice at 24 h post-surgery were collected as controls. Data are expressed as mean ± SD (n = 3–5 per group). *, *P*<0.01 versus *FoxM1 Tg*. (**C**) Lung wet/dry weight ratio. At 24 h post-surgery, lung tissues were collected and dried at 60°C for 3 days. Data are expressed as mean ± SD (n = 4). *, *P*<0.05 versus WT.

To determine whether FoxM1 expression is sufficient to promote endothelial repair following lung injury induced by sepsis, we employed the *FoxM1 Tg* mice in which expression of human *FoxM1* transgene is under the control of the -800-base pair Rosa26 promoter [25]. As shown in **Fig. 1A**, FoxM1 is prominently expressed in lung tissue from *FoxM1 Tg* mice but weakly in WT lungs. At various times following CLP challenge, we determined alterations in lung vascular injury by assessing EBA extravasation, a measure of vascular permeability [26], [28]. *FoxM1 Tg* mice exhibited increase of lung vascular permeability at 12 h post-CLP challenge similar to WT mice (**Fig. 1B**). Lung vascular permeability in *FoxM1 Tg* mice was rapidly restored at 24 h post-CLP challenge and returned to levels similar to baseline seen in sham-operated control mice at 48 h whereas WT lungs remained leaking. Accordingly, lung edema as determined by lung wet/dry weight ratio was resolved in *FoxM1 Tg* mice at 24 h post-CLP challenge in contrast to WT lungs (**Fig. 1C**).

### Accelerated Resolution of Lung Inflammation in *FoxM1 Tg* Mice

**Figure 2:**
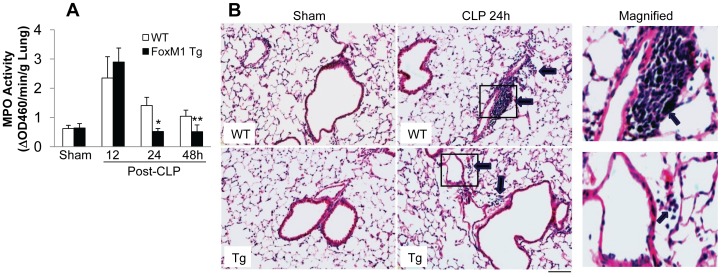
Accelerated resolution of lung inflammation in *FoxM1 Tg* mice. (**A**) MPO activities in lung tissues. Lung tissues at indicated times post-CLP challenge were collected for MPO activity determination. Lung tissues from sham-operated mice at 24 h post-surgery were collected as controls. Data are expressed as mean ± SD (n = 3–5). *, *P*<0.001 versus WT; **, *P*<0.05 versus WT. (**B**) Representative micrographs of H & E staining of lung sections. At 24 h post-surgery, lungs were fixed for sectioning and H & E staining. Arrows indicate perivascular leukocyte infiltration. Scale bar, 50 µm.

We next assessed lung inflammation by measuring MPO activity, an indicator of neutrophil infiltration [18], [27]. As shown in **Fig. 2A**, *FoxM1 Tg* lungs exhibited similar MPO activity at 12 h post-CLP challenge as WT lungs. At 24 h post-CLP challenge, MPO activity in *FoxM1 Tg* lungs was returned to basal levels seen in sham-operated controls whereas it remained elevated in WT lungs. WT lungs exhibited greater MPO activity even at 48 h post-CLP challenge. H & E staining of lung sections revealed greater leukocyte sequestration in WT lungs at 24 h post-CLP challenge compared to *FoxM1 Tg* lungs (**Fig. 2B**).

**Figure 3:**
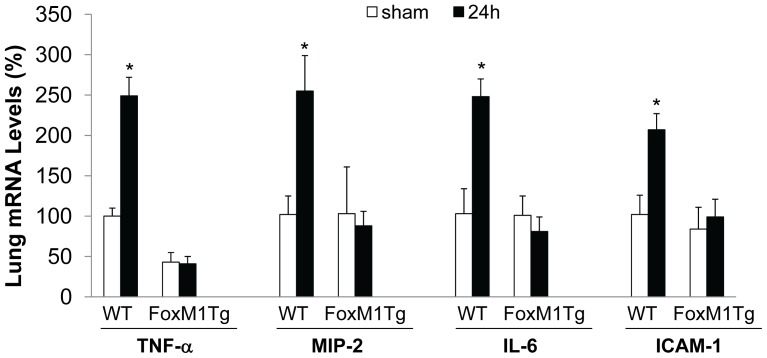
Normalized expression of proinflammatory cytokines and adhesion molecule in *FoxM1 Tg* lungs at 24 h post-CLP. RNA were isolated from lungs collected at 24 h post-surgery and QRT-PCR analysis were employed to assess the expression levels. Data are expressed as mean ± SD (n = 3–4). *, *P*<0.05 versus WT-sham.

To further determine the effects of FoxM1 expression on resolution of lung inflammation, we assessed the expression of proinflammatory cytokines and adhesion molecules. As shown in **Fig. 3**, at 24 h post-CLP challenge, the expression levels of TNF-α, MIP-2 and IL-6 as well as ICAM-1 were returned to basal levels seen in sham-operated controls whereas they were remained elevated, consistent with the concept that overexpression of FoxM1 accelerates the resolution of lung inflammation.

### Marked Increase of Survival of *FoxM1 Tg* Mice

**Figure 4:**
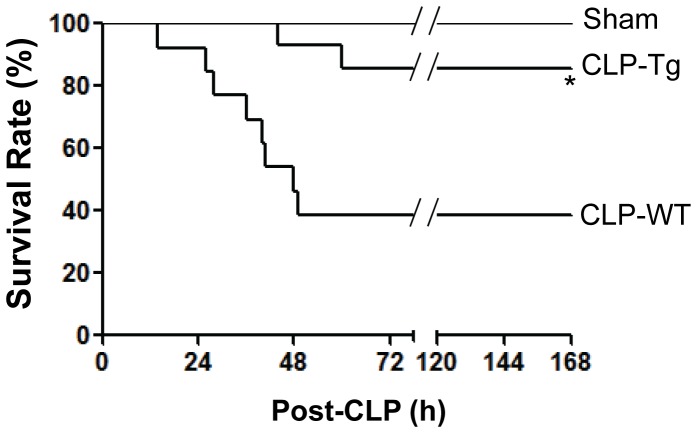
Increased survival of *FoxM1 Tg* mice following CLP challenge. 3 month old mice were monitored for 7 days to determine the survival rate following CLP challenge (n = 13 WT and 14 *FoxM1 Tg*). Sham-operated mice (n = 5 WT or *FoxM1 Tg*) were also monitored for survival. *, *P*<0.001 versus CLP-WT. Tg, *FoxM1 Tg*.

To determine the physiological significance of accelerated vascular repair and resolution of lung inflammation by overexpression of FoxM1, we monitored the survival rate of *FoxM1 Tg* mice following CLP challenge. As shown in **Fig. 4**, more than 60% of WT mice died within 72 h post-CLP challenge whereas only 15% of *FoxM1 Tg* mice died in the same period. None of the sham-operated mice died.

### Rapid Induction of Endothelial Cell Proliferation and Expression of Genes Essential for Cell Cycle Progression in *FoxM1 Tg* Lungs Following CLP Challenge

**Figure 5:**
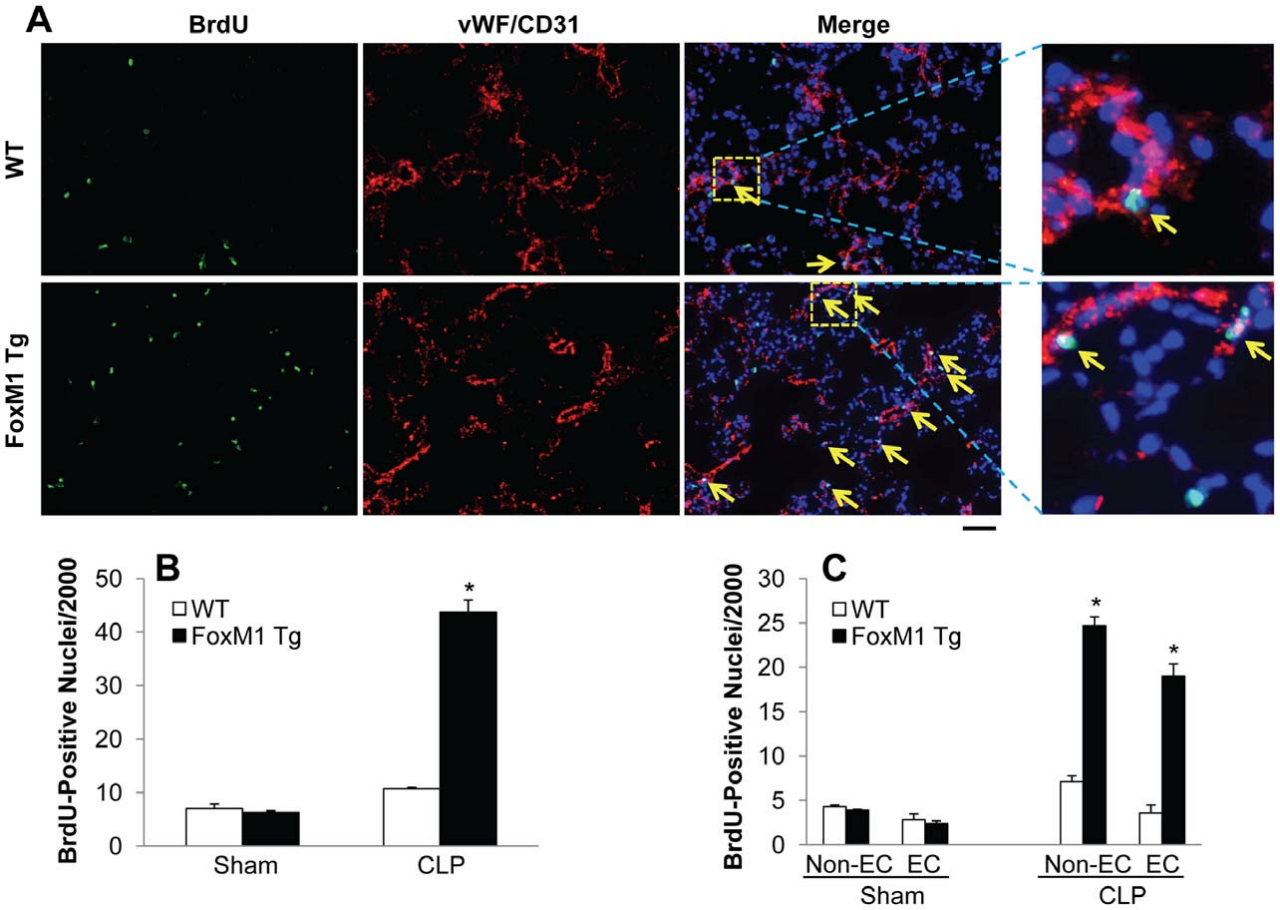
FoxM1-induced endothelial cell proliferation in *FoxM1 Tg* lungs following CLP challenge. (**A**) Representative micrographs of immunofluorescent staining. Lung tissues were collected at 24 h post-CLP challenge, sectioned and immunostained with anti-BrdU (green) and anti-vWF and CD31 (red) antibodies. Nuclei were counterstained with DAPI (blue). Arrows indicate proliferating EC. Scale bar, 50 µm. (**B**) Quantification of BrdU-positive nuclei. Data are expressed as mean ± SD (n = 4 per group). *, *P*<0.001 versus WT. (**C**) Quantification of BrdU-positive EC (vWF^+^ or CD31^+^) and non-EC (vWF^-^ or CD31^−^). BrdU-positive EC were quantified in small vessels (diameter ≤ 100 µm) and capillaries. Data are expressed as mean ± SD (n = 4). *P*<0.001 versus WT.

Our previous study has demonstrated FoxM1-mediated endothelial regeneration is a critical component of the mechanisms of endothelial repair following LPS-induced vascular injury [18]. Thus, we next determined whether FoxM1 expression induces endothelial proliferation thereby promotes endothelial repair. The proliferated cells were labeled by BrdU. Overexpression of FoxM1 resulted in a marked induction of cell proliferation in *FoxM1 Tg* lungs at 24 h post-CLP challenge compared to WT lungs (**Fig. 5A and B**). Quantification of BrdU-positive EC (expressing either CD31 mainly in large vessels or vWF in capillaries) revealed that expression of FoxM1 induced a marked increase of EC proliferation in *FoxM1 Tg* lungs at 24 h post-CLP challenge whereas EC proliferation was minimal at the same period in WT lungs (**Fig. 5A and C**).

**Figure 6:**
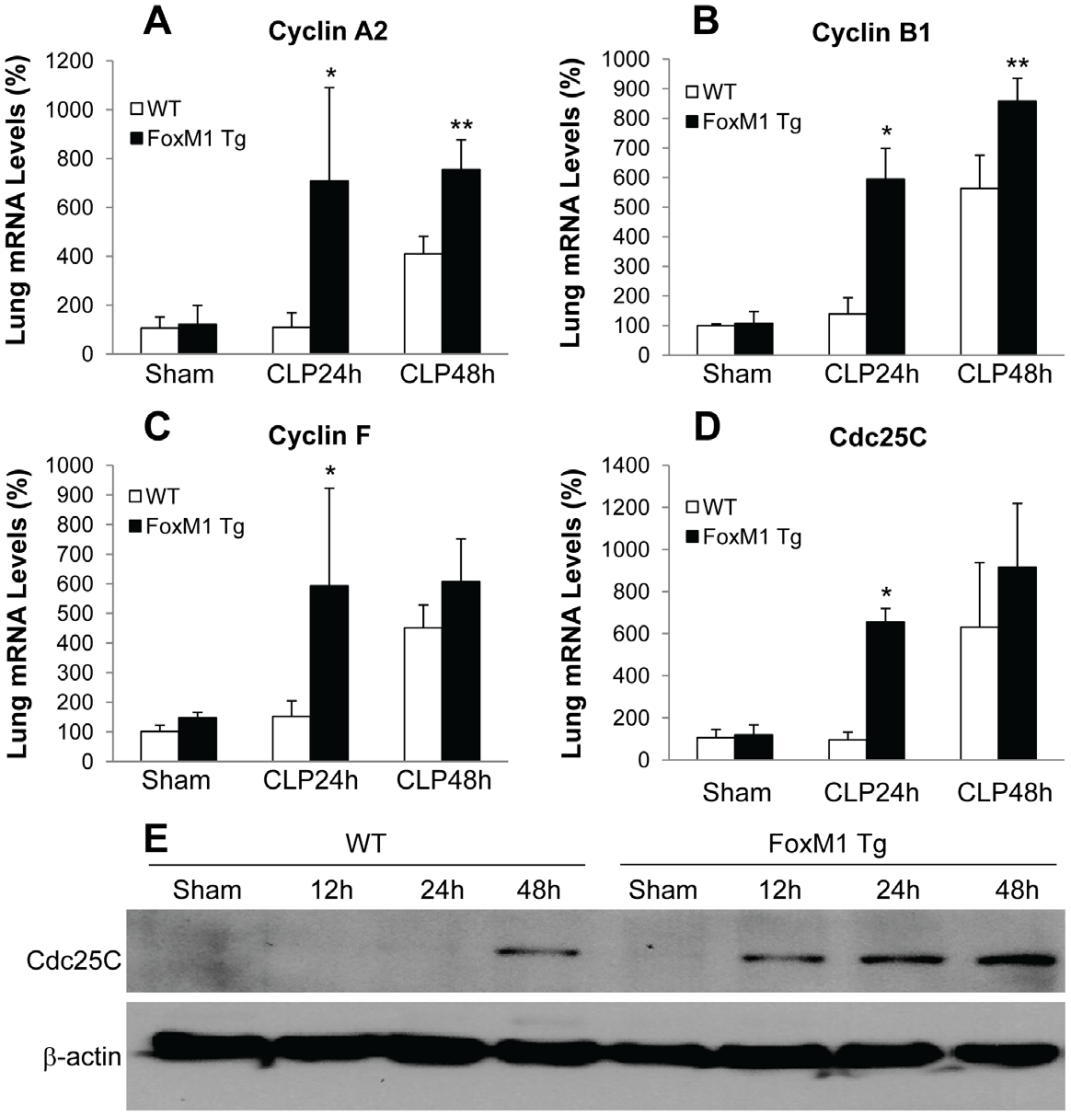
Early induction of expression of FoxM1 target genes essential for cell cycle progression in *FoxM1 Tg* lungs. (**A**–**D**) QRT-PCR analysis of expression of FoxM1 target genes. Lung tissues were collected at indicated times post-CLP challenge or 24 h post-sham operation for RNA isolation and QRT-PCR analysis. Data are expressed as mean ± SD (n = 3–5 per group). *, *P*<0.001 versus WT; **, *P*<0.05 versus WT. (**E**) Representative Western blotting demonstrating FoxM1-mediated induction of Cdc25C protein expression. Lung tissues were collected at various times post surgery and lysed for examination of Cdc25C protein levels by Western blotting. The same membrane was blotted with anti-β-actin as a loading control. The experiment was repeated three times with similar data.

**Figure 7:**
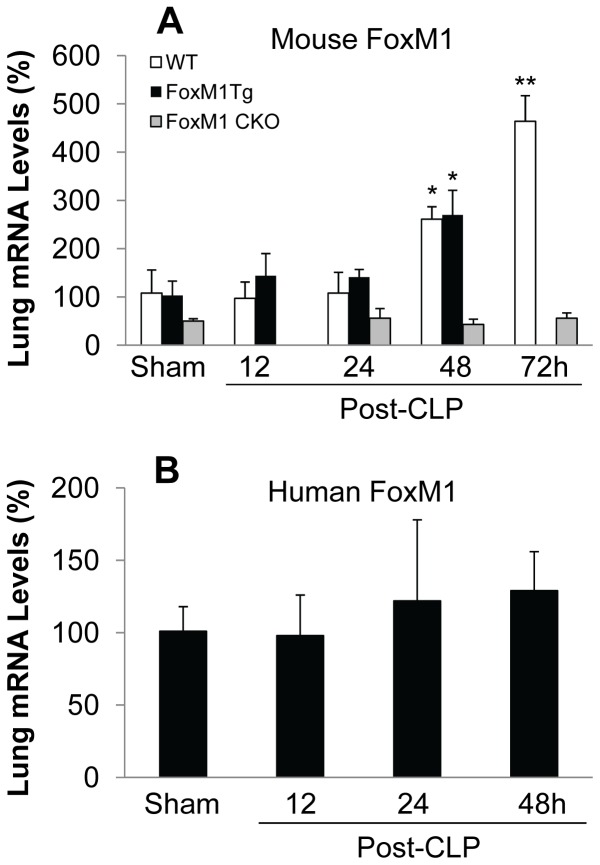
Time course of FoxM1 expression in lungs following CLP challenge. Lung tissues were collected at indicated times post-CLP challenge or 24 h post-sham surgery for RNA isolation and QRT-PCR analysis. Expression of endogenous mouse FoxM1 was assessed with the primers specific with mouse FoxM1 (**A**) whereas expression of the transgene was analyzed with the primers specific with human FoxM1 (**B**). Data are expressed as mean ± SD (n = 3–5 per times). *, *P*<0.01 versus Sham; **, P<0.001. Mouse FoxM1 was similarly induced in WT or *FoxM1 Tg* lungs following CLP challenge but not in *FoxM1 CKO* lungs. Human FoxM1 was constitutively expressed in *FoxM1 Tg* lungs at various times post-CLP.

To investigate the molecular basis of FoxM1-induced cell proliferation, we examined the expression of FoxM1 target genes essential for cell proliferation during endothelial repair [18]. QRT-PCR analysis showed that increased expression of FoxM1 induced expression of cyclins and Cdc25C in *FoxM1 Tg* lungs at 24 h post-CLP challenge whereas the expression of these genes were at basal levels in WT lungs (**Fig. 6A–D**). At 48 h post-CLP challenge, the expression of these genes were also induced in WT lungs, consistent with marked induction of FoxM1 expression in WT lungs at 48 h post-CLP challenge (**Fig. 7A**). Western blotting also revealed a rapid induction of Cdc25C protein expression as early as 12 h post-CLP challenge in *FoxM1 Tg* lungs whereas it was not induced until 48 h post-CLP challenge in WT lungs (**Fig. 6E**).

### Impaired Endothelial Repair in *FoxM1 CKO* Lungs Following CLP Challenge

We have shown that FoxM1 is mainly induced in lung endothelial cells following LPS challenge (18). Similarly, we observed a marked increase of FoxM1 expression in WT lungs at 48 and 72 h post-CLP (**Fig. 7A**). However, FoxM1 induction was completely inhibited in the lungs of *FoxM1 CKO* mice with *Tie2-Cre*-mediated EC-restricted disruption of *FoxM1* (18, 19) following CLP challenge. These data suggest that FoxM1 is predominantly induced in lung EC not other cell types following CLP-induced lung injury.

**Figure 8:**
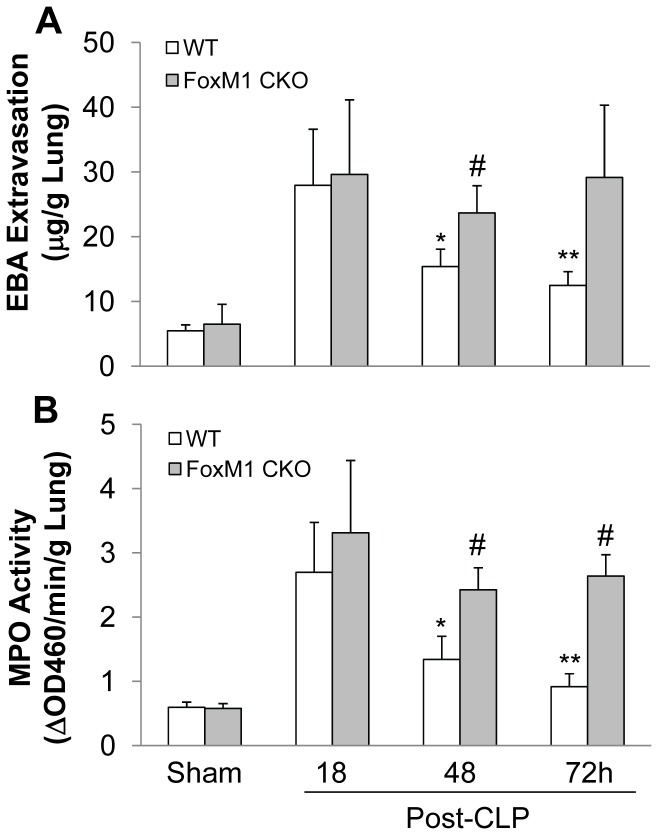
Impaired endothelial repair in *FoxM1 CKO* lungs following CLP challenge. (**A**) EBA extravasation assay demonstrating sustained vascular leakiness in *FoxM1 CKO* lungs. Data are expressed as mean ± SD (n = 4–5). *, *P*<0.01 versus WT-18 h; **, *P*<0.005 versus WT-18 h; #, *P*>0.5 versus *FoxM1 CKO*-18 h. (**B**) Persistent increase of MPO activity in *FoxM1 CKO* lungs following CLP challenge. Data are expressed as mean ± SD (n = 4–5). *, *P*<0.01 versus WT-18 h; **, *P*<0.005 versus WT-18 h; #, *P*>0.5 versus *FoxM1 CKO*-18 h.

We next determined whether FoxM1 expression in EC is indispensable for endothelial repair. As shown in **Fig. 8A**, both WT and *FoxM1 CKO* mice exhibited similar increases of lung vascular permeability at 18 h post-CLP challenge. At late time points (48 h and 72 h), lung vascular permeability in WT mice was markedly reduced whereas it remained elevated in *FoxM1 CKO* mice at levels similar to peak injury. Similarly, we observed a sustained increase of MPO activity in *FoxM1 CKO* lungs at 48 h and 72 h post-CLP challenge whereas MPO activity in WT lungs was drastically decreased at 48 h post-CLP challenge and returned to levels similar to baseline seen in sham-operated controls at 72 h post-CLP challenge (**Fig. 8B**).

## Discussion

We have identified the necessary and sufficient role of FoxM1 in promoting endothelial repair and resolution of lung inflammation in a clinically relevant model of sepsis. We showed that transgenic expression of FoxM1 resulted in rapid recovery of vascular integrity and resolution of lung inflammation following CLP-induced polymicrobial sepsis. *FoxM1 Tg* mice exhibited a marked increase of survival. Mechanistically, we observed early induction of FoxM1 target genes essential for cell cycle progression and resulting endothelial proliferation in *FoxM1 Tg* lungs following CLP challenge. Additionally, selective deletion of FoxM1 in EC resulted in defective endothelial repair in *FoxM1 CKO* mice following CLP-induced lung vascular injury. Together, these data demonstrate the critical role of FoxM1 in mediating endothelial repair following lung vascular injury induced by sepsis.

Endothelial repair requires endothelial regeneration and subsequent re-annealing of the endothelial cell-cell contacts to restore the characteristic restrictive endothelial barrier function [2], [8], [29]. Our previous studies have demonstrated the essential role of FoxM1 in regulating endothelial proliferation and re-annealing of the endothelial adherens junctions complexes employing the *FoxM1 CKO* mice [18], [19]. This study further demonstrated the sufficient role of FoxM1 in promoting endothelial regeneration and restoration of endothelial barrier integrity. We observed similar increases of lung vascular permeability and inflammation in WT and *FoxM1 Tg* mice in the initial responses to CLP challenge. However, transgenic expression of FoxM1 promoted rapid recovery of vascular integrity and resolution of inflammation. These data suggest increased FoxM1 expression has little effects on the initial injury responses to CLP challenge but plays an important role in promoting endothelial repair.

We observed a marked increase of expression of FoxM1 target genes including cyclins A2, B1, and F as well Cdc25C in *FoxM1 Tg* lungs at 24 h post-CLP challenge. Early induction of expression of these genes correlates with the increased rate of cell proliferation in *FoxM1 Tg* lungs. In future studies, it would be interesting to determine whether induction of these target genes is responsible for FoxM1-mediated endothelial proliferation and thereby endothelial repair. siRNA-mediated knockdown of one or more of the cyclins may be helpful to address this questions. Intriguingly, increased expression of FoxM1 in *FoxM1 Tg* lungs at basal failed to induce expression of these genes and cell proliferation. As a transcription factor, FoxM1 location in the nucleus is essential for its transcription activity [16], [30], [31]. It has been shown that ectopic expression of FoxM1 leads to increased FoxM1 protein levels in the cytoplasm but not in the nucleus at basal. Following injury, proliferative stimuli induce FoxM1 translocation into the nucleus to activate expression of target genes and resulting cell proliferation. Thus, it is likely that in response to lung injury induced by CLP challenge, FoxM1 expressed in *FoxM1 Tg* lungs is quickly translocated into the nucleus and subsequently activates endothelial proliferation to promote endothelial repair.

In agreement with our observation, previous study has also shown that overexpression of FoxM1 induces expression of genes regulating cell cycle progression, and promotes cell proliferation including lung endothelial cells and epithelial cells in a different model of lung injury induced by butylated hydroxytoluene challenge [25]. Furthermore, our study demonstrate for the first time the physiological significance of overexpression of FoxM1 and resultant endothelial regeneration in the mechanism of lung endothelial repair following inflammatory vascular injury.

Consistent with rapid restoration of endothelial barrier integrity in *FoxM1 Tg* lungs, we observed rapid resolution of lung inflammation as demonstrated by decreased MPO activity and leukocytes sequestration as well as normalized expression of proinflammatory genes and adhesion molecule which were at baseline levels at 24 h post-CLP. These data support the generalized concept that the endothelium monolayer helps to maintain the anti-inflammatory state of microvascular bed and injured endothelium promotes inflammation.

Our data showed a marked increase of FoxM1 expression in WT lungs at 48 and 72 h post-CLP challenge. However, the induction of FoxM1 expression was not seen in the early phase of injury following CLP challenge, consistent with its important role in mediating endothelial regeneration and barrier repair. Accordingly, we observed significant induction of expression of FoxM1 target genes cyclins A2, B1 and F as well as Cdc25C in WT lungs at 48 h post-CLP.

A limitation with the *FoxM1 Tg* mice in the current study is that expression of human *FoxM1* transgene is under the control of the Rosa26 promoter [25]. Given that human FoxM1 transgene is expressed in most of the cell types, FoxM1 expression in cells other than EC may also contribute to the enhanced endothelial repair in *FoxM1 Tg* mice following CLP challenge. Employing the *FoxM1 CKO* mice, our data have shown the importance of FoxM1 induction in EC in mediating endothelial repair. Inhibition of FoxM1 expression impaired recovery of endothelial barrier integrity and resolution of lung inflammation as indicated by sustained increases of EBA extravasation and MPO activity in *FoxM1 CKO* lungs following CLP challenge. Consistent with this observation, we have shown that FoxM1 is markedly induced in WT lungs only in the repair phase following LPS challenge [18]. The induced FoxM1 expression is critical for endothelial regeneration and barrier repair following LPS-induced lung vascular injury [18]. Thus, it is likely that FoxM1 expression in EC is the common critical mediator of endothelial repair following lung vascular injury induced by various sepsis challenges. Targeting FoxM1 to acutely activate the endothelial repair program may represent an effective approach to restore the endothelial integrity and reverse leaking microvessels for the treatment of ALI/ARDS.
